# Tuberculous meningitis diagnosis and treatment: classic approaches and high-throughput pathways

**DOI:** 10.3389/fimmu.2024.1543009

**Published:** 2025-01-10

**Authors:** Fangbo Lin

**Affiliations:** Rehabilitation Medicine Department, The Affiliated Changsha Hospital of Xiangya School of Medicine, Central South University (The First Hospital of Changsha, Changsha, China

**Keywords:** tuberculous meningitis, high-throughput, omics, sequencing, diagnosis, treatment

## Abstract

Tuberculous meningitis (TBM), a severe form of non-purulent meningitis caused by *Mycobacterium tuberculosis* (Mtb), is the most critical extrapulmonary tuberculosis (TB) manifestation, with a 30–40% mortality rate despite available treatment. The absence of distinctive clinical symptoms and effective diagnostic tools complicates early detection. Recent advancements in nucleic acid detection, genomics, metabolomics, and proteomics have led to novel diagnostic approaches, improving sensitivity and specificity. This review focuses on nucleic acid-based methods, including Xpert Ultra, metagenomic next-generation sequencing (mNGS), and single-cell sequencing of whole brain Tissue, alongside the diagnostic potential of metabolomic and proteomic biomarkers. By evaluating the technical features, diagnostic accuracy, and clinical applicability, this review aims to inform the optimization of TBM diagnostic strategies and explores the integration and clinical translation of multi-omics technologies.

## 1 Introduction

Tuberculous meningitis (TBM), caused by *Mycobacterium tuberculosis* (Mtb), is a fatal form of non-purulent meningitis and one of the deadliest types of extrapulmonary tuberculosis (TB). Despite the availability of anti-TB treatment, the mortality rate for TBM exceeds 30% (1), with even higher rates in HIV co-infected individuals (2). Early diagnosis and treatment are essential, but challenges persist due to TBM’s nonspecific clinical signs and the low Mtb load in cerebrospinal fluid (CSF). Traditional diagnostic methods, such as acid-fast staining, have low sensitivity, while Mtb culture is time-consuming (3–7). Recent advancements in diagnostics, such as Xpert Ultra and mNGS, have improved sensitivity and shortened diagnostic delays (8–10). Additionally, metabolomic and proteomic biomarkers, such as elevated citrulline, lactate, apoB, and NELL2, hold promise for diagnosis and differential diagnosis (11, 12).

**Figure 1:**
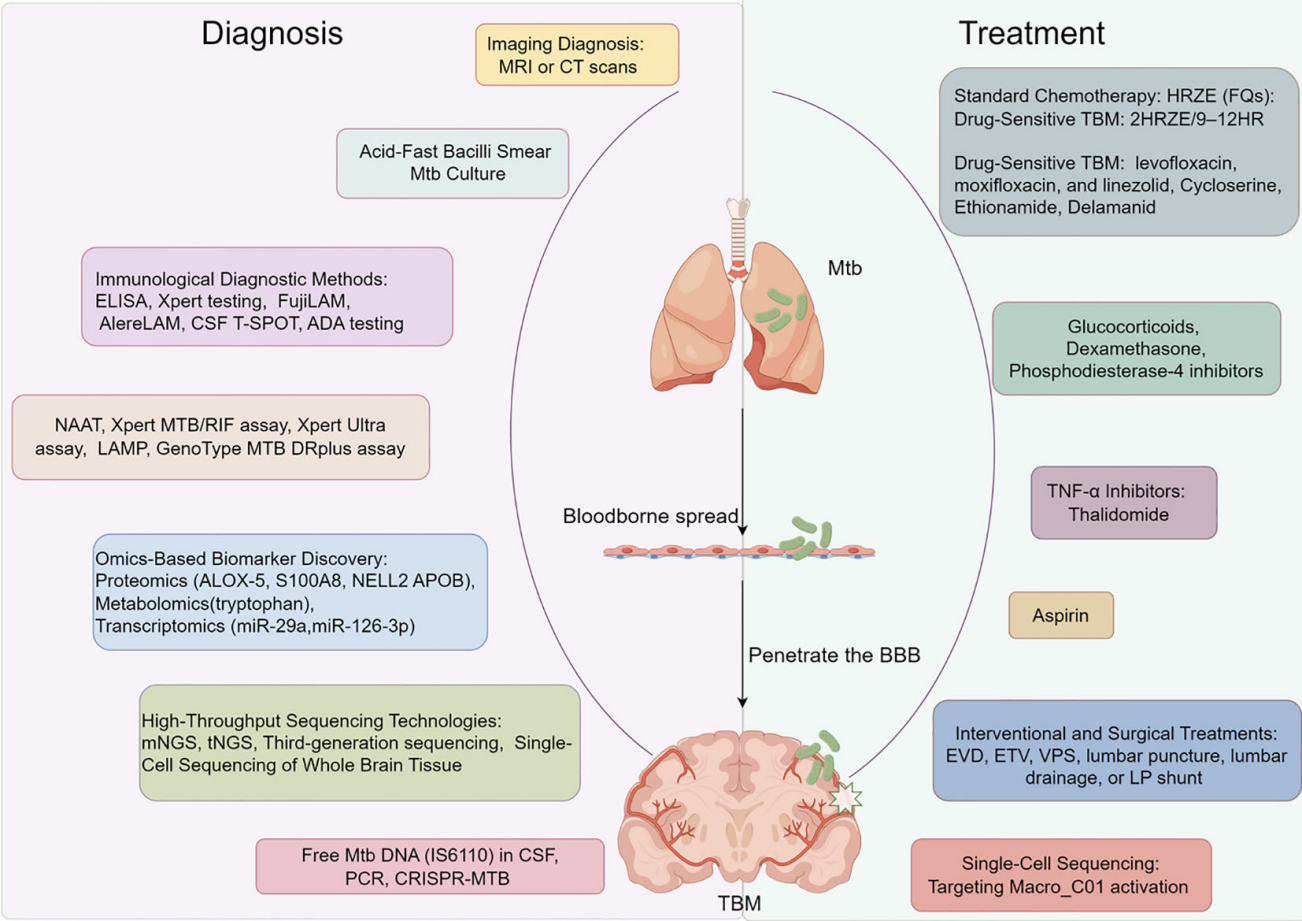
Tuberculous meningitis diagnosis and treatment.

Furthermore, single-cell sequencing and omics technologies offer new insights into TBM pathogenesis and potential therapeutic targets (13, 14). This review provides an overview of recent advances in TBM diagnostics and therapeutics, covering nucleic acid detection, omics-based biomarker discovery, pharmacological therapies, and adjunctive strategies, offering a comprehensive reference for clinical practice (**Figure 1**).

## 2 Diagnosis of TBM

### 2.1 Imaging diagnosis of TBM

Radiologically, TBM presents with basal cistern and meningeal enhancement, brain infarction, hydrocephalus, and tuberculomas, with basal meningeal enhancement being a key feature (15). Contrast-enhanced MRI or CT should be performed before and after treatment to monitor progression. Thoracic CT and other imaging techniques help identify active TB lesions (16, 17). Pathologically, TBM includes inflammatory exudates, granulomas, and vasculitis, with 77% of patients showing cerebral arteritis, leading to infarction and hydrocephalus (2, 18). Rare cases, such as brain tuberculomas post-BCG vaccination, emphasize vigilance in vaccinated infants (19). Some patients exhibit pituitary adenoma-like lesions with headaches and vision loss. Combining imaging and histopathology is essential for accurate diagnosis (20).

### 2.2 Cerebrospinal fluid diagnostic methods

The gold standard for TBM diagnosis is detecting Mtb in CSF. However, due to low bacterial load, conventional bacteriological tests show limited sensitivity (3, 4). Enhancing sensitivity remains a major research focus.

#### 2.2.1 Acid-fast bacilli smear and Mtb culture

Acid-fast staining is rapid and cost-effective but has low sensitivity (<20%), occasionally near 0% (2, 21). Modified techniques, such as centrifuging larger CSF volumes and using fluorescence microscopy, have improved positivity rates to 60% (21). Mtb culture remains the “gold standard.” Liquid cultures are about 10% more sensitive than solid cultures but are prone to cross-contamination, while solid cultures have low sensitivity (4.3%) and take up to 56 days (22) (**Table 1**). Repeated CSF sampling and modified acid-fast staining can improve results, though challenges persist (23, 24). Fluorescence microscopy, including LED fluorescence, offers higher sensitivity at lower costs (16), though resource limitations hinder its widespread use (22).

**Table 1:** Comparison of diagnostic methods for tuberculous meningitis.

Diagnostic Method	Sensitivity	Specificity	Cost	Time Requirements
Acid-Fast Bacilli Smear	<20%, sometimes near 0%	High	Low	Rapid (minutes to hours)
Modified Acid-Fast Smear	Up to 60%	High	Low	Rapid
Mtb Culture	4.3% (solid), ∼14.3% (liquid)	High	Moderate to High	Up to 56 days
LAM Detection	75% (lateral flow)	Variable	Low	Rapid
IGRA	76%	88%	Moderate	Variable
ADA Testing	89%	91%	Low	Rapid
Xpert MTB/RIF	63% (culture ref.), 82% (large-volume CSF)	>90%	High	2 hours
Xpert Ultra	60-90%	>90%	High	2 hours
LAMP	76%	99%	Low	Rapid
mNGS	61%	98%	High	Variable
tNGS	81.8% for CNS infections	76.9% *vs*. 100% for others	High	∼24 hours
Omics-Based Biomarkers	Varies	Varies	Variable	Variable

Mycobacterium tuberculosis (Mtb) culture; Lipoarabinomannan (LAM) Detection; Gamma-interferon release assays (IGRAs); Adenosine deaminase (ADA) testing; metagenomic next-generation sequencing (mNGS); Loop-mediated isothermal amplification (LAMP); Targeted next-generation sequencing (tNGS).

#### 2.2.2 Immunological diagnostic methods

CSF antigen-antibody detection has long been studied but is hindered by poor sensitivity and specificity of many tests. A 2015 study reported sensitivities of 75% and 43% for detecting lipoarabinomannan (LAM) with lateral flow and ELISA methods, respectively, while Xpert testing showed 100% sensitivity (25). Although FujiLAM is more sensitive than AlereLAM in urine (26), its diagnostic value in CSF is unclear. Gamma-interferon release assays (IGRAs), such as CSF T-SPOT, detect TBM infection by measuring gamma-interferon levels, with sensitivity of 76% and specificity of 88%. However, it requires large CSF volumes (>6 mL) and has a 15% indeterminate rate (27). Adenosine deaminase (ADA) testing in CSF shows 89% sensitivity and 91% specificity of TBM, but ADA levels in bacterial and viral meningitis complicate interpretation due to unclear diagnostic cut-offs (28).

#### 2.2.3 Advances in nucleic acid amplification technologies

Nucleic acid amplification tests (NAAT) are essential for TBM diagnosis, enhancing sensitivity (82%) and specificity (99%) versus culture, and 68% sensitivity with 98% specificity against clinical diagnosis (29, 30). The World Health Organization (WHO)-recommended Xpert MTB/RIF detects TB and rifampin resistance within 2 hours (31), with 63% sensitivity versus culture, increasing to 82% with large-volume CSF (32, 33). Xpert Ultra, with a sevenfold lower detection threshold (22, 31), achieves 60–90% sensitivity and >90% specificity (34, 35) but is limited by high cost and equipment requirements (36, 37). Loop-mediated isothermal amplification (LAMP) offers 76% sensitivity and 99% specificity (38), reaching 88% sensitivity and 100% specificity when multiple targets are used, though contamination risks persist (39). The GenoType MTB DRplus assay detects Mtb and resistance genes with 33% sensitivity and 98% specificity, constrained by complexity and cost (40).

#### 2.2.4 Omics-based biomarker discovery for TBM diagnosis

Advances in omics technologies offer promising avenues for identifying TBM-specific biomarkers, as CSF may harbor distinct markers due to the unique pathogenic mechanisms of TBM.

(і) Proteomics: Limited studies include Kataria et al. (41), who identified 19 differential proteins with ALOX-5 as a potential marker; Ou et al. (42), who confirmed the association of S100A8 and APOB using ELISA; and Mu et al. (43), who found 111 differential proteins, highlighting NELL2 and APOB. These studies require larger, more diverse samples for validation.

(іі) Metabolomics: Li et al. (44) identified 25 metabolites differing between TBM and viral meningitis using 1H-NMR, while another study (45) reported significant changes in CSF and blood metabolites, particularly reduced tryptophan correlated with prognosis. Multicenter validation is needed.

(ііі) Transcriptomics: MicroRNAs such as miR-29a (46) and miR-126-3p (47) show potential as diagnostic markers for TBM, but further research is necessary across different diagnoses.

#### 2.2.5 Application of high-throughput sequencing technologies

##### 2.2.5.1 Metagenomic next-generation sequencing

mNGS sequences genetic material using random primers, filtering out host DNA to identify microorganisms present (39, 48). It outperforms traditional methods in detecting Mtb, viruses, anaerobes, and fungi, particularly in cases missed by Xpert Ultra or culture (48). A 2019 study reported mNGS sensitivity of 66.67% for TBM, higher than smear (33.33%), PCR (25%), and culture (8.33%) (3), with meta-analyses showing 61% sensitivity and 98% specificity (49). However, mNGS may detect contaminants causing false positives (50–52). Low Mtb sequences in CSF due to DNA extraction issues can be improved with optimized centrifugation (50, 53), with optimized centrifugation improving sensitivity (53). Combining mNGS with other methods may enhance detection rates (3, 6, 54, 55).

##### 2.2.5.2 Targeted next-generation sequencing

tNGS combines targeted PCR amplification with high-throughput sequencing, focusing on specific nucleic acid regions to reduce interference from human genes and background flora, thus decreasing detection costs and time (around 24 hours) (56, 57). However, tNGS can only detect predefined targets, limiting its ability to identify unknown pathogens and relying on clinician judgment (58). DeeplexMyc-TB, introduced in 2019, uses tNGS to identify 18 drug resistance genes in the Mtb complex and detect resistance to multiple anti-tuberculosis drugs (59). tNGS has demonstrated high efficiency in detecting drug-resistant TB (60). Although its effectiveness in TBM diagnosis remains untested, studies show tNGS achieves 81.8% sensitivity for CNS infections, surpassing culture and smear (13.6%), though with lower specificity (76.9% *vs*. 100%) (61). In one case, tNGS successfully detected Mtb in a patient with negative mNGS results for bronchoalveolar lavage fluid and lung tissue, confirming secondary tissue-associated pneumonia. The patient improved significantly after anti-TB therapy (62).

##### 2.2.5.3 Third-generation sequencing (long-read sequencing)

Third-generation sequencing enables direct analysis of single DNA or RNA molecules without the need for fragmentation or amplification, providing longer reads ideal for studying high-repeat, GC-rich Mtb sequences (63). The PacBio SMRT platform detects fluorescence signals during nucleotide incorporation, aiding in the identification of Mtb genomic methylation sites and revealing lineage-specific and resistance-associated methylation patterns (64–68). These profiles correlate with transcription levels, nitrogen metabolism, and protein interactions (66, 67). Oxford Nanopore Technologies (ONT) uses nanopore sequencing, which detects changes in electrical current as nucleic acids pass through pores. The MinION platform offers rapid detection of Mtb resistance genes. Despite higher error rates, ONT’s calibration aligns its results with second-generation sequencing, achieving 94.8% sensitivity and 98.0% specificity with shorter detection times (69–75).

##### 2.2.5.4 Single-cell sequencing of whole brain tissue

Using 10X Genomics, Zhang et al. identified 15 cell types in whole brain tissue and observed inflammatory transcriptional changes across them. Stat1 and IRF1 were found to mediate inflammatory responses in macrophages and microglia, while decreased oxidative phosphorylation in neurons correlated with neurodegeneration in TBM patients. Reduced Frmd4a in ependymal cells was linked to hydrocephalus and neurodegeneration (13). Through motif enrichment analysis (miReact), they constructed miRNA-mRNA networks, including immune-inflammatory networks in macrophages and microglia, oxidative phosphorylation networks in neurons, and ion and protein transport networks in ependymal cells. qRT-PCR and RNA scope revealed significantly elevated miR-21a-3p levels in TBM brain tissue compared to normal tissue, suggesting its potential as a diagnostic biomarker (14).

The implementation and application of high-throughput technologies can significantly enhance diagnostic accuracy and speed, thereby improving patient outcomes. However, simplified procedures and user-friendly interfaces can further facilitate the adoption of these technologies by non-experts, making their integration into clinical workflows more effective.

#### 2.2.6 Other molecular biological detection methods

Emerging molecular detection methods show promise in diagnosing TBM but require further validation for clinical use. Li et al. (76) found that free Mtb DNA (IS6110) in CSF exhibited a 56.5% sensitivity in 46 clinically diagnosed TBM patients, surpassing smear (2.2%), culture (13%), and Xpert (23.9%). Shao et al. (77) reported a 93.3% sensitivity for free Mtb DNA in 84 suspected TBM cases, matching Xpert Ultra and outperforming culture (13.3%). Li et al. (78) used digital PCR to detect IS6110 in CSF from 101 HIV-negative TBM patients, achieving a sensitivity of 70%, higher than Xpert (30%). Ai et al. (79) applied CRISPR technology for diagnosing pulmonary and extrapulmonary TB, requiring smaller samples and shorter detection times. In a study of 27 TBM patients, CRISPR-MTB sensitivity was 73%, outperforming Xpert (54%) and culture (25%). Larger studies are necessary to validate these methods for wider clinical adoption.

#### 2.2.7 Comparative analysis of diagnostic methods and the potential of combined approaches

Diagnostic methods for TBM each have distinct strengths and limitations. Traditional techniques like acid-fast smear and culture are widely accessible but suffer from low sensitivity and long turnaround times. Nucleic acid amplification methods (e.g., Xpert MTB/RIF, mNGS) offer higher sensitivity and rapid results but are costly and require specialized equipment (80). Immunological assays provide quick diagnostics but often lack specificity. Omics-based approaches (proteomics, metabolomics, transcriptomics) show promise for identifying specific biomarkers but need further validation (81). Integrating multiple diagnostic modalities, such as combining nucleic acid amplification with omics biomarkers, may enhance sensitivity and specificity, enabling earlier detection and better differentiation from other forms of meningitis. Future research should focus on validating combined strategies in diverse clinical settings to improve TBM diagnosis.

## 3 Treatment of TBM

### 3.1 Standard chemotherapy

Treatment for drug-sensitive TBM typically includes a combination of rifampin (R), isoniazid (H), ethambutol (E), and pyrazinamide (Z) (HRZE), supplemented by fluoroquinolones (FQs), with a treatment duration of over nine months (82). Increasingly, adjunctive immunotherapy and interventional therapies are used. Key considerations include selecting drugs with good blood-brain barrier (BBB) permeability, adjusting dosages, and combining FQs to optimize outcomes. For multidrug-resistant (MDR) TBM, regimens must include at least four effective first- or second-line drugs, with attention to BBB penetration and prolonged treatment (83).

#### 3.1.1 Drug-sensitive TBM

WHO recommends an initial 2-month intensive HRZE regimen, followed by 9–12 months of HR consolidation therapy (2HRZE/9–12HR) (84). However, this approach, based on pulmonary TB protocols, does not fully address BBB drug penetration. Pyrazinamide, with excellent BBB penetration, is crucial, and higher doses may optimize therapy (85). Isoniazid also effectively crosses the BBB, though high-dose efficacy remains unclear, with a recommended dose of 300–600 mg/day (86). Rifampin is essential, but its CSF concentration often falls below the minimum inhibitory concentration (MIC). High-dose rifampin (20–35 mg/kg) may improve efficacy, though its impact on survival needs further study (84, 85, 87, 88). Recent research suggests that combining high-dose rifampin with levofloxacin during the intensive phase does not significantly improve survival, though it raises rifampin CSF concentrations (89). Additionally, other treatment strategies can be classified based on drug-sensitive TBM. For instance, adjunctive therapies such as glucocorticoids and aspirin are primarily recommended for drug-sensitive cases to reduce inflammation and prevent neurological complications (90). These treatments are integrated with standard chemotherapy to enhance patient outcomes. Furthermore, immunomodulatory agents like TNF-α inhibitors may also be considered, although their use is more cautiously approached in drug-sensitive TBM due to potential side effects (91).

#### 3.1.2 Drug-resistant TBM

WHO guidelines for MDR and rifampin-resistant (RR-TB) TBM emphasize DST-guided therapy and the use of drugs with high BBB permeability (92, 93). Key agents include levofloxacin, moxifloxacin, and linezolid, which have good CNS penetration (85, 94). Linezolid improves clinical parameters such as body temperature, CSF leukocyte count, and treatment success rates, although its safety and efficacy in resistant TBM require further investigation (95, 96). Cycloserine and ethionamide are viable alternatives to ethambutol but are associated with neurotoxicity and gastrointestinal side effects (97). Delamanid offers superior survival outcomes in drug-resistant TBM due to excellent CNS penetration (85). In contrast, drugs like para-aminosalicylic acid and ethambutol have poor permeability and limited effectiveness (94). Moreover, certain treatment strategies are more effective for drug-resistant TBM. For example, the use of second-line anti-TB drugs such as bedaquiline and linezolid is crucial in managing MDR-TBM. These drugs, often combined with fluoroquinolones and other agents with good BBB penetration, provide a tailored approach to overcoming resistance (98). Additionally, adjunctive therapies like aspirin and glucocorticoids may be utilized to mitigate inflammatory responses, although their roles are more prominently defined in drug-sensitive TBM (99). The differentiation in treatment approaches underscores the importance of accurate TBM classification to optimize therapeutic efficacy.

### 3.2 Glucocorticoids

Glucocorticoids, primarily dexamethasone, reduce inflammation, repair BBB damage, and decrease brain edema, improving survival in HIV-negative TBM patient (85, 100, 101). Hydrocortisone is used for hyponatremia due to cerebral salt wasting, however, they do not lower long-term neurological sequelae risks or clearly prevent strokes (85, 101). Combining glucocorticoids with anti-TB therapy reduces acute-phase mortality and neurological damage, though CNS damage may persist after tapering dexamethasone (102, 103). Phosphodiesterase-4 inhibitors (102) and thalidomide has shown some efficacy as adjunct therapy in selected cases but is limited by significant side effects (103, 104). Although extending dexamethasone treatment may improve outcomes, CNS damage risk persists after dose tapering or discontinuation (103). Most guidelines recommend adjunctive corticosteroids in the acute phase, but long-term efficacy and safety warrant further study (102, 103).

### 3.3 Tumor necrosis factor-α inhibitors

TNF-α plays a key role in TBM pathogenesis by activating macrophages and forming caseous granulomas. Thalidomide, a TNF-α inhibitor, reduces inflammation and improves survival in TBM animal models, halving mortality rates. However, its teratogenicity and potential to activate T-cells limit its use in pregnant women and broader clinical application (105, 106).

### 3.4 Aspirin

Aspirin provides antithrombotic and anti-inflammatory effects by inhibiting cyclooxygenase and reducing prostaglandin release, lowering stroke risk in TBM (100). Misra et al. (107) found that while aspirin did not significantly reduce stroke incidence, it reduced mortality. Higher doses improved therapeutic outcomes (108, 109). Yadav (110) suggested combining aspirin with corticosteroids to reduce TBM mortality, forming a basis for Phase III trials. Rizvi et al. (111) showed that while aspirin combined with anti-TB regimens did not lower mortality, it significantly reduced stroke risk without increasing adverse events compared to placebo. Current evidence supports aspirin’s efficacy in mitigating stroke risk, but more studies are needed to confirm long-term benefits when combined with corticosteroids (107).

### 3.5 Interventional and surgical treatments

TBM patients often develop hydrocephalus and brain edema (112). Surgical interventions like external ventricular drainage (EVD), third ventriculostomy (ETV), or ventriculoperitoneal shunting (VPS) can relieve intracranial pressure when conventional treatments fail (113). Other options include lumbar puncture, lumbar drainage, or lumbar-peritoneal (LP) shunt to avoid cranial surgery. Endoscopic third ventriculostomy is suitable for non-communicating hydrocephalus. Loan et al. (113) reported a 1-month mortality rate of 33.3%-61.9% in HIV-positive adults undergoing VPS for TBM, though further studies are needed. Kamat et al. found VPS blockage rates of 27.5% in pediatric and 25.5% in adult TBM patients. High CSF protein concentration (2.94 g/L in blocked cases *vs*. 1.76 g/L in non-blocked cases) was identified as a risk factor, emphasizing the need to reduce CSF protein levels before VPS (114).

### 3.6 Single-cell sequencing reveals potential therapeutic targets

Single-cell transcriptomics by Mo et al. identified 33 monocyte populations in CSF and PBMCs of children with TBM, highlighting distinct myeloid clusters and CD4/CD8 T-cell subsets with unique effector functions. Complement-activated microglial cells (Macro_C01) were linked to neuroinflammatory responses associated with persistent meningitis, amplifying inflammatory signaling through interactions with CD4_C04 subsets. Targeting Macro_C01 activation has been suggested as a therapeutic approach for pediatric TBM. Elevated C1Q, CRP, and cytokines (TNF-α, IL-6) in CSF further indicate their potential as TBM diagnostic biomarkers (115).

## 4 Conclusion

TBM remains highly lethal and disabling due to its complex pathophysiology and the lack of sensitive, specific diagnostics, which delay early diagnosis and treatment. Recent advancements in laboratory diagnostics, including nucleic acid-based methods, proteomics, and metabolomics, have enhanced diagnostic sensitivity and specificity despite technical challenges. Therapeutically, combining traditional anti-tuberculous treatments with adjunctive therapies has improved survival rates and reduced complications. Surgical innovations like ventriculoperitoneal shunting and third ventriculostomy effectively manage TBM-related hydrocephalus and intracranial hypertension. Additionally, single-cell sequencing and transcriptomics are identifying therapeutic targets, advancing precision medicine. Integrating these diagnostic and therapeutic strategies with public health initiatives is essential to reduce TBM’s global burden and improve patient outcomes.
